# Supplementary material for: Novel quantitative trait loci conferring resistance to root-knot nematodes in lima bean (Phaseolus lunatus)
Source: Mol Breed. 2026 Sep 24;46(10):97. doi: 10.1007/s11032-026-01719-9 (PMC13612800; doi:10.1007/s11032-026-01719-9)
Supplement: Supplementary file 1 [file 11032_2026_1719_MOESM1_ESM.docx]

**Supplementary Figure 1:** RIL01, RIL02 and RIL03 Whole Genome LOD Peaks

(a.) depicts a whole genome representation of the LOD peaks associated with respective traits in the RIL01 population. (b.) depicts a whole genome representation of the LOD peaks associated with respective traits in the RIL02 population. Finally, (c.) depicts a whole genome representation of LOD peaks associated with respective traits in the RIL03 population.

a.

b.

c.

**Supplemental Figure 2:** Lima bean root systems of RIL02 and RIL03 (modified Bridge and Page scale) A score of 10 indicates complete root death and a score of 0 indicates a completely clean root system. Images of these scores were not available.

| Score = 1   | Score = 2   | Score = 3   |
| --- | --- | --- |
| Score = 4   | Score = 5   | Score = 6   |
| Score = 7   | Score = 8   | Score = 9   |

**Supplemental Figure 3**: Mean phenotypic values for two genotypic classes (i.e. parental haplotypes) at SNPs in the major QTL on chromosome 5. SNP(s) chosen based on overlapping (in the same bin) SNPs between RIL01, RIL02 and RIL03 populations (Chr05_38009108 – Chr05_38080061). P values calculated from a one way ANOVA test in RStudio. Bars represent the average phenotypic value of populations with the respective allele at selected SNP on chromosome 5.

**Supplemental Table 1:** Data table for RIL02 (Henderson x PI 256874) population. ERS: eggs/root system. EGR: eggs/gram of root.

*Parental Phenotypes*

|  | *M.incognita* ERS (mean) | *M. incognita* EGR (mean) | *M. javanica* ERS (mean) | *M. javanica* EGR (mean) | *M. incognita* gall mean (0-10 scale) | *M. javanica* gall mean (0-10 scale) |
| --- | --- | --- | --- | --- | --- | --- |
| Henderson | 79,667 | 5,610 | 89,167 | 3,417 | 6.33 | 6.20 |
| PI 256874 | 10,667 | 175 | 22,000 | 430 | 3.22 | 0.4 |

*Phenotype Class 1 (lines with the PI phenotype) - 38 lines*

|  | *M.incognita* ERS | *M. incognita* EGR | *M. javanica* ERS | *M. javanica* EGR | *M. incognita* gall mean | *M. javanica* gall mean |
| --- | --- | --- | --- | --- | --- | --- |
| Mean | 22,250 | 754 | 23,556 | 808 | 3.16 | 2.49 |
| Standard Deviation | 19,854 | 664 | 20,019 | 727 |  |  |

*Phenotype Class 2 (lines with Henderson phenotype) - 42 lines*

|  | *M.incognita* ERS | *M. incognita* EGR | *M. javanica* ERS | *M. javanica* EGR | *M. incognita* gall mean | *M. javanica* gall mean |
| --- | --- | --- | --- | --- | --- | --- |
| Mean | 146,453 | 4,303 | 137,313 | 4,082 | 6.39 | 4.43 |
| Standard Deviation | 60,792 | 2,467 | 112,199 | 3,139 |  |  |

*Phenotype class 3: (low M. javanica galling) - 17 lines*

|  | *M.incognita* ERS | *M. incognita* EGR | *M. javanica* ERS | *M. javanica* EGR | *M. incognita* gall mean | *M. javanica* gall mean |
| --- | --- | --- | --- | --- | --- | --- |
| Mean | 106,559 | 5,701 | 56,794 | 3,481 | 5.25 | 2.18 |
| Standard Deviation | 62,006 | 2,622 | 64,045 | 3,430 |  |  |

*Phenotype Class 4 (M. incognita resistance - low galling and low egg counts) - 13 lines*

|  | *M.incognita* ERS | *M. incognita* EGR | *M. javanica* ERS | *M. javanica* EGR | *M. incognita* gall mean | *M. javanica* gall mean |
| --- | --- | --- | --- | --- | --- | --- |
| Mean | 29,128 | 840 | 83,295 | 2,553 | 3.39 | 3.11 |
| Standard Deviation | 15,241 | 501 | 59,260 | 1,309 |  |  |

**Supplemental Table 2:** QTL haplotype effects within RIL03 (PI256874 x L-136). ERS: eggs/root system. EGR: eggs/gram root.

| Line | Donor parent @  *QRk_pl04.1* | Donor parent @  *QRk_pl05.1* | Donor parent @  *QRk_pl10.1* | Has putative resistance QTL? | *M. incognita* mean galling (0-10 scale) | *M. incognit*a mean ERS | *M. incognit*a mean EGR | *M. javanica* mean galling (0-10 scale) | *M. javanica* mean ERS | *M. javanica* mean EGR |
| --- | --- | --- | --- | --- | --- | --- | --- | --- | --- | --- |
| RIL047 | L-136 | PI 256874 | PI 256874 | yes | 4.67 | 6,482 | 25 | 1 | 401,111 | 1,965 |
| RIL063 | L-136 | PI 256874 | PI 256874 | yes | 1.83 | 13,889 | 67 | 0.5 | 111,389 | 1,632 |
| RIL070 | L-136 | PI 256874 | PI 256874 | yes | 0.83 | 236,111 | 710 | 0 | 33,333 | 147 |
| RIL072 | L-136 | PI 256874 | PI 256874 | yes | 3.50 | 42,593 | 170 | 2 | 356,667 | 1,954 |
| RIL081 | L-136 | PI 256874 | PI 256874 | yes | 3.50 | 927 | 4 | 0.5 | 283,333 | 1,691 |
| RIL083 | L-136 | PI 256874 | PI 256874 | yes | 3.50 | 103,704 | 266 | 1.5 | 628,889 | 2,556 |
| RIL054 | Heterozygous/PI 256874 | L-136 | L-136 | no | 8.5 | 1,052,778 | 4,728 | 2.5 | 925,000 | 7,445 |
| RIL071 | PI 256874 | L-136 | L-136 | no | 7.00 | 505,555 | 3,621 | 3.5 | 2,026,667 | 29,067 |
| RIL087 | PI 256874 | L-136 | L-136 | no | 8.00 | 637,963 | 3,718 | 2.5 | 733,334 | 8,975 |
| PI 256874 | PI 256874 | PI 256874 | PI 256874 | partial | 5.92 | 404,167 | 1,628 | 1.3 | 315,370 | 1,594 |
| L-136 | L-136 | L-136 | L-136 | partial | 2.83 | 323,148 | 2,204 | 0 | 494,074 | 2,691 |
| Henderson | Henderson | Henderson | Henderson | no | 9.58 | 750,926 | 4,614 | 5 | 2,100,000 | 18,722 |

**Supplemental Table 3:** F1 phenotype tables for RIL01 (Henderson x L-136), RIL02 (Henderson x PI 256874) and RIL03 (PI 256874 x L-136) populations. Galling: + indicates presence of galling, - indicates absence of galling.

|  | ERS  *M. incognita* | EGR  *M. incognita* | galling  *M. incognita* | ERS  *M. javanica* | EGR  *M. javanica* | galling  *M. javanica* |
| --- | --- | --- | --- | --- | --- | --- |
| Henderson | 563,730 | 5,712 | **+** | 635,079 | 5,926 | **+** |
| L-136 | 162,143 | 1,343 | **-** | 449,603 | 3,431 | **-** |
| PI 256874 | 42,619 | 161 | **-** | 25,238 | 83 | **-** |
| Henderson x L-136 (F1) | 710,159 | 6,177 | **-** | 427,778 | 2,895 | **-** |
| Henderson  x PI 256874 (F1) | 711,667 | 7,005 | **+** | 611,574 | 6,460 | **+** |

|  | ERS  *M. incognita* | EGR  *M. incognita* | galling  *M. incognita* | ERS  *M. javanica* | EGR  *M. javanica* | galling  *M. javanica* |
| --- | --- | --- | --- | --- | --- | --- |
| Henderson | 426,667 | 3,381 | **+** | 339,722 | 3,629 | **+** |
| L-136 | 403,111 | 2,220 | **-** | 1,011,111 | 5,257 | **-** |
| PI 256874 | 31,111 | 104 | **-** | 78,556 | 221 | **-** |
| PI 256874 x L-136 (F1) | 524,723 | 2,000 | **-** | 4,422,444 | 22,638 | **-** |

**Supplemental Table 4:** Flanking sequences for *QRk-pl04.1* and *QRk-pl05.1*

| QTL locus | Left hand marker | Sequence (500 bp, +/- 250 bp) | Right hand marker | Sequence (500 bp, +/- 250 bp) | Interval (kb) |
| --- | --- | --- | --- | --- | --- |
| *QRk-pl04.1* | Chr04_46033436 | TAAAGATTTTATGCTGTATAGATTAAGATACTACCTTAAAATTAATGTCTCTAGTGGCACACTGTAATACATTCTTAATCCTACGAACACCAGCTAGTTAATTCTATGTCTCAACAGTCTCTACCTACCACACCACAACCACCACCAACAAGAACAACAACAGCTATTTCAAGTTCTCTTCCACCCTTTTCTTTTTCACTCTTCTAATTCTTCTTCACTCACCAACATGGCACCCTCAACCCCTCAAGT**[C/A]**TCACCTGCAAAGGTAAACTCAGCCACCCTTTTCTTTTGATATCTCTGTCTATCCGTTGTTTATGATGAACATGGATAGTGGGGTTTTTGTTAATGATGGCATGTGGAACATGAAACAGCTGCAGTGGCATGGGGAGCTGGGGAGCCACTGGTGATGGAGGAAGTGGAAGTGAGTCCTCCCCAACCAATGGAGATTAGGATTAAGGTTGTCTCCACCTCTCTCTGCCGCAGTGATCTCTCTGCTTGGGAAT | Chr04_47051812 | ATCTTTTACATTATCGCCATACTTAAGAAATTTAAAACTTAAATGTGATATTTACGTTTTTTCAATGAAAATTACAAAATTATCTTGATTGAAATTTAATTTAATTTACTCACTATGACAACCTTTTACTGTAGTGTTTGATTCTAGAGATATATTGAAAATGGTATGACCTTTCAAAATAACTGTAGTTTTGATCATATAAACAAAATTAAAATAACTCATGTTCTCACTTTTTCTTTATTTCTATAAA**[A/T]**TTTTGAAAAAAGATATTTAGGGTTAGTATAGAGAAAGAGGCTGTGTAGCAGAGGGCATGTTGATGAAGATAACAGTTACATGATTGCATGATGAGAGCATTAATTGCAGAAGAATATGAATGTCTGCATTATTATTTTGGCAAGGAAGATGATGTAAACACTGTGAAAGATGTGAAAGGGACAGATAACTCCACCATCGCCATAACTCCACATTGATCTACCACTGTAGGGCAAATAAATCATTGAAAAT | 1,018.38 |
| *QRk-pl05.1* | Chr05_38009108 | CTTCTGGTTACCGATTTCGAAAACGTTTTCCTCACCAAAATATATTTTCACAAACATTTTCATTACTTCCTTTTATCTCGCTCACTTCTTGTTTTTTCTTCTCAAACTTTCTTTTTTGCTCATCAAGAAACTGATTGGTCTTCTATTATCTTCGTTTTTTTGTTTCATTCCTCACAGGAATATCTGCATTACAGTTTCAGCTTCTTCCTCTAAACATGCTTTTCATTCTTCATTTCAGACCATTCTTCTTC**[A/T]**CAGAACCGTTTTCGAACCTATTTTTCACTTTTCCTCAGCTCCGAATATTCGGATCATCTGGTCTCATCATGTTGGATGCATTATTCATGCCGAAATTCTATTCAAAGAGGTAATGATGCTTATCTGTTTCTCTTCTTTCATTTTTTTTTCTCTCTTCCTCGTTTTGGATTTTTTTTCTTAAACTAATATATAACAATGGAATTAAATTGGGAAACAGCAAATCACGGTTGAAGTTAATAAATAGGAGGT | Chr05_38270149 | ATCAGCTCTTCTCTACCTCTATGACATTAGTAACAGAGTTGATGAAAGAAGGCAAAGCTCTACTTCCCGCAAAGGCCTCTGCGTCAATATTCAAGTGCTCATATCGTACTTCGATGGTTGGAATATCAAGCCCAACTCTACATGAAACAACAAGCCAAACGAAAACTGAAAAACATGAGACTCAAAAACTCCTACCACTATAGGGAAGAAGAGGTTGTGAAGAAGAGGTAATATACCTGTCAATACGCTC**[T/C]**TGAGCTTCAACAAGAACCTCTCGTTGTCCTCTTCGGCCACTTTGACCAACCTCTCAAGAAGCTTTTGTTTCTGTTGGAAGCCGAGATCAGAAACATCGATTTCGTTGGCAACTCCATGGGATGCTGTCAACAAGCCTTTCCTGAGACGGTTGTAGGTGGGGAGCTTCTCAAGAGCAGCCCATTTGAGAGCTTCTTCGTCATCCTCTTCACGAGAAGACCTTGAAAATGCCTCCACACCACTGTTCCTCCA | 261 |
